# Regional destination attributes that attract domestic tourists: the role of man-made venues for leisure and recreation

**DOI:** 10.1016/j.heliyon.2021.e07383

**Published:** 2021-06-25

**Authors:** Andres Camacho-Murillo, Rukmani Gounder, Sam Richardson

**Affiliations:** a Faculty of Economics, Universidad Externado de Colombia, Calle 12 No. 1-17 Este, Bogotá, Colombia; b School of Economics and Finance, Massey University, Tennent Drive Palmerston North 4474, New Zealand

**Keywords:** Domestic tourism, Man-made attractions, Tourism for leisure and recreation, Mixed logit model, Factor analysis

## Abstract

This paper examines the influence of man-made attractions for leisure and recreation on domestic tourists' preferences amongst regional destinations, and the moderating role of these attractions on the negative effect of distance on tourists' choices. A mixed multinomial logit model is employed for 368 cities in Colombia grouped into 28 provinces. Factor analysis is utilised to identify the latent variable that groups several man-made attractions for leisure and recreation. Results show that domestic tourists' choices of a regional destination increase as the number of man-made attractions for leisure and recreation rises, although there is taste heterogeneity between tourists explained by their city of origin. Findings also show that the decline in domestic tourists' preferences for a regional destination due to increases in travel distance can be lessened through the construction and/or enhancement of man-made venues for leisure and recreation in the destination; a strategy that can serve to reduce monetary poverty in distant destinations that have attributes to attract tourists.

## 1 Introduction

Domestic tourism is a significant form of travel in various countries that recorded between 5 and 6 billion domestic trips in 2015 (UNWTO, 2016), and accounted for 71% of total global travel and tourism spending in 2019 (WTTC, 2020). It is a source of revenues that can contribute to the regional economic development within the countries. Domestic tourism for leisure and recreational activities has been investigated from diverse perspectives, including residents' choices among regional destinations as a function of regions' attributes (Huybers, 2003; Nicolau and Mas, 2006). Huybers (2003) linked the empirical findings to Lancaster, 1966, Lancaster, 1971 model of characteristics, which suggests that the properties of the goods (the destination attributes in this case) are the object of consumers' utility.

There are several regional destination attributes that attract domestic tourists, including climate (Becken, 2013; Bujosa and Rosselló, 2013; Denstadli et al., 2011; Eugenio-Martin and Campos-Soria, 2010a; Marcussen, 2011), beaches (Marrocu and Paci, 2013), and world heritage sites (Patuelli et al., 2013). Man-made attractions for visitor's leisure and recreation have received less attention in the literature, in spite that several venues that domestic tourists visit during their trip belong to this category of attributes. Museums and restaurants (Marrocu and Paci, 2013) and aerial trams (Hearne and Salinas, 2002) have been analysed as some of the attributes that attract domestic tourists to a regional destination. This paper extends the literature by including further man-made venues that can influence domestic tourists' choices, such as theme parks and shopping malls, following Swarbrooke (2002)'s list of man-made attractions for leisure and recreation.

It has been found that domestic tourist's trips for leisure purposes can last, in average, a minimum of 1.6 days (Yang et al., 2011) or more than four days (Grigolon et al., 2014) depending on the season. It is plausible, therefore, that several man-made attractions can be jointly visited by domestic tourists during their regional visitation. Ultimately, leisure tourists are encouraged to maximise their trip experience in the destination by visiting diverse venues and sites for recreational purposes, which will become an important part of tourists' memories and experiences. This paper analyses clustering patterns between man-made venues for leisure and recreation as a contribution to the existing literature on the link between domestic tourists' choices and the attributes of tourism destinations.

Based on Train (1998), domestic tourists with the same observed characteristics are likely to have different tastes for each destination attribute that influences their choices. Domestic tourists' heterogeneity has been recognised by Nicolau and Mas (2006) around physical, cultural and interpersonal motivations. The size of the city where domestic tourists travel from is seen as one of the key sources of heterogeneity examined in this paper. Domestic tourists from cities with more than fifty thousand inhabitants have been found to travel more as compared to tourists from municipalities with less than fifty thousand inhabitants (Alegre et al., 2013). It is unknown whether domestic tourists from large cities tend to travel more than domestic tourists from smaller cities motivated by the number of man-made attractions in the destination. This is an important aspect to evaluate for policy initiatives that promote the construction and/or enhancement of venues for leisure activities.

The distance between residents' city of origin and their tourist destination is another key determinant studied in domestic tourism (Huybers, 2003; Marrocu and Paci, 2013; Monfort et al., 2010; Nelson, 2013; Priego et al., 2015). Nicolau and Mas (2006) and Nicolau (2010) confirmed the distance decay theory in the demand for domestic tourism, although found that domestic tourists' motivations and inertial behaviour can moderate the negative effects of distance on domestic tourists' choices. This paper critically examines whether the construction and/or enhancement of man-made attractions can moderate the negative effect of distance on tourists' preferences. Based on Nelson (2013), it is likely that domestic tourists travel further away as the number of man-made venues in the destination increases. In developing countries where poor regions with tourism potential are located far from the main cities, the understanding of this issue will be crucial for policy initiatives.

The aim of this paper is to analyse the role of destination attributes on domestic tourists' preferences, with emphasis on man-made attractions for visitors' leisure and recreation. The paper seeks to examine, first, whether domestic tourists' choices of a province are more likely if the average number of man-made attractions (per square kilometre) rises. Second, whether there is heterogeneity between domestic tourists around the mean coefficient of man-made attractions that can be explained by the size of domestic tourists' city of origin. Thus, the demographic characteristics of domestic tourists by specific city categories indicate the level of influence based on their choices. Third, whether an increase in the number of man-made visitor attractions in a province can potentially offset the negative effect of distance on domestic tourists' preferences for that province.

To model the relationship between destination attributes and domestic tourists' preferences amongst provincial destinations, and the pattern of heterogeneity between domestic tourists around destination attributes, the Mixed Logit model is employed (Train, 2009). Data on domestic tourists are sourced from the National Survey of Domestic Tourism Spending published by Colombia's Administrative Department of Statistics. Data on destination attributes are taken from diverse secondary sources. Factor analysis is utilised to construct the latent variable that clusters the set of man-made attractions in the regional destinations; a latent variable that is then used to examine the effects of man-made attractions on the choices of domestic tourists.

The rest of this paper shows, first, a literature review on the influence of man-made attractions and distance on domestic tourists' preferences. Then, the paper presents the model of domestic tourists' choices, followed by a discussion of data. After discussing the empirical results, the paper presents important conclusions and implications of the findings.

## 2 Literature review

### 2.1 Man-made attractions and domestic tourists' choices

There are several destination attributes studied in the literature that influence the choices of domestic tourists, including man-made visitor attractions. An attraction is a feature, venue or activity in a place that provides appropriate facilities and services for visitors (locals and tourists), it is manageable, and can be offered with or without entry fees (Swarbrooke, 2002). Marrocu and Paci (2013) investigated the impact of restaurants and museums on the demand for domestic tourism in the provinces of Italy in 2009. Using the gravity model of trade and a Spatial Autoregressive (SAR) model, the authors' findings suggest that the number of restaurants (with at least one Michelin star) positively influences the trip to a domestic tourist destination. The number of museums partially explains domestic tourists' choices in Italy (Marrocu and Paci, 2013). For Massidda and Etzo (2012), the promotion of cultural activities associated with museums can significantly attract tourists, although the effect is lower than the impact of other cultural factors.

Hearne and Salinas (2002) studied the preferences of domestic and foreign tourists visiting Poás Volcano in Costa Rica to undertake ecotourism activities. One of the attributes included in this study was the presence of aerial trams (also called gondola lifts), which are “aerial public transit technology propelled from above by cables” (Alshalalfah et al., 2012, p. 253). Using the conditional logit model, Hearne and Salinas' study finds that the visual motivation associated with aerial trams is a significant determinant for domestic tourists to visit Poás Volcano in Costa Rica, even more than for foreign visitors.

Theme parks and shopping malls are attractions within Swarbrooke's (2002) list of man-made venues that can be included in empirical studies, as they also tend to be visited by domestic tourists during their trip. Theme parks offer combined experiences of learning, nature contact (animals, plants), thrill (roller coasters, water rides), and leisure (general entertainment, restaurants, and shops) that captivate visitors (McClung, 2000). When there are several theme parks within a region, cluster patterns of visitation tend to occur (Fodness and Milner, 2000). Sun and Uysal (1994) noted that after the openness of Walt Disney World and its three theme parks (the Magic Kingdom, EPCOT Centre, and the Disney-MGM Studio), the total number of tourists in Florida (United States) grew significantly.

Shopping malls represent a more recent concept of man-made visitor attractions, as they “…offer services and facilities for entertainment, shopping, eating, drinking and other aspects of leisure” (Stevens, 2003, p. 285). For Leask (2008) and Swarbrooke (2002), shopping malls are not seen solely as a support service for visitors' leisure and recreation. All visitors are influenced by the idea of having diverse activities to do in one venue, including shopping, socialising, eating, and entertaining, among other activities, which are offered by shopping malls (Grube-Cavers and Carvajal-Sánchez, 2014). Thus, a visit to shopping malls in a destination during a domestic trip can satisfice the taste of each visitor within a family and/or a group of friends.

Man-made attractions built to attract visitors can be the main reason for a trip; although for some domestic tourists, they can just be a complement to the journey's purpose of visiting other attractions, including nature-base attractions, venues built for purposes other than attracting visitors, and special events (Swarbrooke, 2002). On this basis, empirical studies have shown that domestic tourists' choices of a regional destination are influenced by climate (Bujosa and Rosselló, 2013), nature-based attractions such as beaches (Bujosa and Rosselló, 2013; De-la-Mata & Llano-Verduras, 2012; Marrocu and Paci, 2013) and heritage sites (Patuelli et al., 2013; Priego et al., 2015). Thus, correlation patterns between regional attributes that serve to attract domestic tourists are expected to arise.

### 2.2 Tourists' city of origin: a source of heterogeneity

Following discrete choice theory (Hensher and Greene, 2003; Train, 1998), differences in tastes between domestic tourists around each regional attribute are likely, and can be explained by individuals' characteristics. Domestic tourists' city of origin can be one of the characteristics that influences domestic tourists' trip to a destination for leisure activities. Alegre et al. (2013) found that residents from comparatively large municipalities (with more than fifty thousand people) tend to travel more for tourism purposes than residents from smaller municipalities (with less than fifty thousand people). Notwithstanding, residents from smaller cities could be more motivated than residents from large cities to travel to regions where the number of man-made attractions is comparatively large. The rationale is that large cities generally offer several entertainment options for residents, including museums, theatres, quality restaurants, shopping malls, and other man-made attractions (Maitland and Newman, 2009) compared to smaller cities.

Doubts remain on whether all tourists from smaller cities are equally motivated to visit those regions that offer more man-made attractions for leisure and recreation. Based on Hensher and Greene (2003), differences between tourists from different city categories may still exist due to certain individual sociodemographic characteristics that mark taste diversity. In studies on domestic tourism participation, the likelihood of travelling for holidays activities has been found to be higher in married domestic tourists than those divorced or separated (Eugenio-Martin and Campos-Soria, 2010b). However, when one of the trip motivations is to enjoy man-made attractions as an attribute of the regional destination, it may occur that married domestic tourists from smaller cities are less concerned of this attraction category than other tourists within the marital status category. Little evidence exists on this matter in the domestic tourism literature.

Some literature provides close evidence supporting the idea that married tourists' preferences regarding man-made attractions for leisure (or the like) differ from the preferences of single and divorced tourists for the same group of attractions (regardless the category of tourists' city of origin). Bojanic (1992) shows that married residents from the United States of America are less concerned of doing activities of adventure, excitement, and nightlife than single residents (in a bachelor stage) during their international trip. Similarly, Kaynak et al. (1996) find that, in Dublin (Ireland), married people are more attracted by restful and physically refreshing destination attributes, while single tourists tend to value more the experiences associated with excitement, nature, fun, amongst other attributes. For the Spanish case, Rojo-Ramos et al. (2021) find that amongst the marital status category, single tourists are more oriented toward adventure tourism activities. Based on Nicolau (2010), the spirit of variety-seeking for man-made attractions (in tourists from relatively smaller cities) is likely more pronounced in single and divorced tourists than married tourists. Single and divorced residents tend not to be worried about children in their travel plans as noted by Bojanic (1992) for the case of families in the United States.

### 2.3 Distance and tourists' choices: the moderating role of man-made attractions

The distance between domestic tourists' region of origin and their destination is another important determinant in the literature of domestic tourism demand (Gálvez et al., 2014; Huybers, 2003; Marrocu and Paci, 2013; Monfort et al., 2010; Priego et al., 2015). The relationship between tourists' preferences for a region and the distance travelled from the home region to the destination has followed the distance decay theory; this is, “the demand for any good or service should decline exponentially as distance increases” (McKercher et al., 2008, p. 208).

The association between the trip and distance travelled can reflect several spatial conditions in decision-making, of which the time constraint is one. Morley (1992) and Hsu et al. (2013) show tourists' utility as a function of trip length constrained by vacation time. According to Balestrino (2011) and the classical model of consumption, paid-work and leisure time by Becker (1965), people are encouraged to optimise their time between work and leisure activities. Distance itself can also reflect budget constraints associated with displacement costs between residents' city of origin and destination (Bujosa and Rosselló, 2013; Priego et al., 2015). Land transport is the most appealing for tourists, with cars being the most frequent (Rothengatter, 2010; cited by Aguiló et al., 2012), due to competitive travel costs compared with the cost of other transport modes (Moyano et al., 2016). Finally, distance can mirror tourists' regionalist behaviour from a social distance perspective (Thyne and Zins, 2003). Based on Gray's (2004) concept of regionalism, residents tend to prefer nearby destinations due to social reasons, including cultural compatibility with the region, devotion and pride in their own regions, and the desire for fostering their communities.

Few studies have examined the interaction term of distance with explanatory factors that affect domestic tourists' choices. Nicolau and Mas (2006) and Nicolau (2010) show that domestic tourists' motivations and inertial behaviour, respectively, moderate the effects of distance on visitors' choices. If man-made attractions can influence domestic tourists' preferences, but the distance effect counteracts this, a positive compensation effect is anticipated in the interaction effect. The reason is that the residents of a city are willing to travel to distant destinations, as they seek to visit venues that attract tourists (Nelson, 2013), of which man-made attractions for leisure can be included.

## 3 Model and data

### 3.1 Model

To examine the role of destination attributes on domestic tourists' choices, and the key related research hypotheses, the Mixed Logit model for micro-level data is employed. This is a generalisation of Mc Fadden's (1973) Conditional Logit model, in which an individuals' expected utility is a function of the attributes of alternatives rather than individuals' characteristics. These attributes represent the “pull factors” that attract tourists (the travel object); although there are also sociological factors, “push factors”, that explain tourists' predisposition to travel (Dann, 1977). The Mixed Logit model has the capability of capturing individuals' heterogeneity around the attributes of each alternative included in the model (Train, 1998). Following Train, 1998, Train, 2009, the random utility model applied in this paper shows that domestic tourists' utility is $U_{ni}=v_{ni}+\varepsilon_{ni}$, where $v_{ni}$ accounts for the observed proportion of domestic tourists' utility from visiting province i for leisure activities, and $\varepsilon_{ni}$ is the error term that is identically and independently Gumbel distributed. The choice probability model is as follows:

(1) $$P_{ni}=\int \left(\frac{e^{v_{ni}}}{\sum_{j}e^{v_{nj}}}\right)\cdot f\left(\beta_{n}|b,\eta_{n}\right)d\beta_{n}$$

Where $P_{ni}$ accounts for the probability that a domestic tourist n travels to province i (against the alternatives j). The term $v_{ni}=\beta_{n}^{\prime }X_{ni}$, where $X_{ni}$ is a vector of attributes in province i, and $\beta_{n}$ is a vector of coefficients to estimate. Here, $\beta_{n}=b^{\prime }X_{ni}+\eta_{n}^{\prime }X_{ni}$, b is domestic tourists' mean taste, and $\eta_{n}$ is the unobserved domestic tourists' taste (the standard deviation from the mean). An advantage of the Mixed Logit model (compared to the conditional logit model) is that the assumption of independence from irrelevant alternatives (IIA) no longer holds (Train, 1998); therefore, any pattern of substitution between alternatives (provinces) can be exhibited (Train, 2009).

The travel distance between domestic tourists' city of origin and his/her destination and the climate in the destination are two attributes that can influence domestic tourists' observed utility (Nicolau and Mas, 2006). Following Swarbrooke's (2002) typology of visitor attractions, nature-based attractions (such as beaches), man-made attractions originally built for reasons other than recreational activities (such as war memorials), and man-made attractions for leisure activities are other destination attributes that attract domestic tourists. The latter category can include theme parks, gondola lifts, museums, restaurants, and shopping malls following Swarbrooke (2002) and data availability.

Each of the man-made visitor attractions mentioned above can enter the model as a separate attribute that influences domestic tourists' choices in its own unique way. However, it is highly likely that several man-made attractions are jointly visited by domestic tourists during their length of stay, which has been found around 1.6 days (Yang et al., 2011); 2.6 days (Garín-Muñoz, 2009); between 3 to 4.5 days (McKercher, 1998), or more depending on the season (Grigolon et al., 2014). Following Fabrigar et al. (1999) and Costello and Osborne (2005), factor analysis is used in this paper to create a latent variable that captures the correlation pattern among man-made attractions in the destinations (hereafter referred to as *MANMADE*) while reducing the dimensionality of data.

The provincial attributes that influence domestic tourists' choices can be added to domestic tourists' observed utility function in Eq. (1) as follows:

(2) $$v_{ni}=\beta_{0}+\beta_{1n}DISTANCE_{i}+\beta_{2n}TEMPERA_{i}+\beta_{3n}TEMPERA_{i}^{2}+\beta_{4n}BEACH_{i}+\beta_{5n}MEMORIAL_{i}+\beta_{6n}MANMADE_{i}$$

Where: $DISTANCE_{ni}$ is the distance in kilometres (km) to travel to province i from tourist's city of residence. Travel time could be more appropriate than travel distance; however, the latter is used in this study due to the lack of travel time precision that occurs when tourists travel across the complex mountain system of Colombia (the Andes). $TEMPERA_{ni}$ is average temperature in the destination province i. The choice of a regional destination by the tourist n is based on the average temperature of his/her city of residence and the average temperature of the region to visit. Following Bujosa and Rosselló (2013), the average temperature is also squared to identify the turning point, $TEMPERA_{ni}^{2}$. As temperature cannot be administered, it is treated as a destination attribute and separate from visitor attractions (Swarbrooke, 2002). $BEACH_{i}$ is the average number of beaches in province i (per square kilometre). $MEMORIAL_{i}$ is the average number of war memorials alluding to the independence war from Spain in province i (per square kilometre). $MANMADE_{i}$ is the latent variable for the number of man-made venues in province i (per square kilometre). By expressing each visitor attraction in per square-kilometre units, the concentration of each attraction in the province visited is captured. The more concentrated an attraction is in a region, the more specialized the region is in the provision of that attraction for locals and tourists.

**Figure 1:**
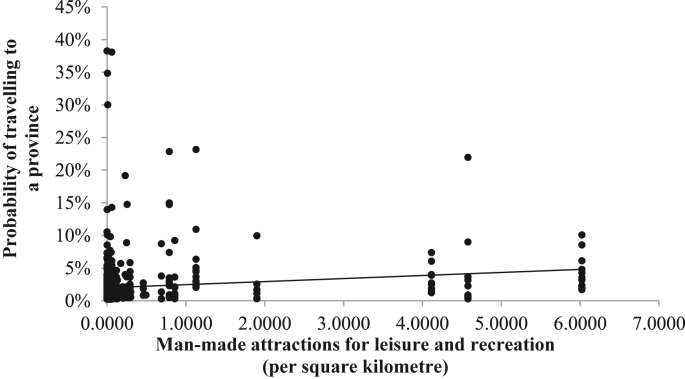
Domestic tourists' taste heterogeneity around the mean coefficient of Man-Made attractions per square kilometre.

The estimated coefficients of Eq. (2) (from $\beta_{1n}$ to $\beta_{6n}$) are used to analyse whether domestic tourists' choice of a destination is more likely due to the presence of each destination attribute in that destination. Particular attention is paid to the estimated coefficient $\beta_{6n}$ to examine the first hypothesis of this paper; this is, whether domestic tourists' choices of a provincial destination are more likely if the average number of man-made attractions ($MANMADE_{i}$) is greater. It is believed that regions with a larger number of manmade attractions are mostly visited; however, this is not always the case. E.g., Bogotá, Medellín, and Cali are cities in Colombia with the largest numbers of man-made attractions; however, they are in the 3rd, 5^th^, and 14^th^ place of cities that receive domestic tourists the most. Figure 1 shows the strong heterogeneity that exists between tourists' choices of a regional destination motivated by the number of man-made attractions (per square kilometre).

A potential reverse-causality between the variable $MANMADE_{i}$ and domestic tourists' choices of a regional destination may exist. Arguably, this bidirectional causality is likely not strong in several destination cities of Colombia, as the man-made venues included in this study are mainly enjoyed by the locals. However, in cities that are highly oriented toward tourism activities, this reversal causality could be evident. Based on Chiang and Wainwright (2005), the man-made venues included in this study as a predictor of domestic tourists' preferences are taken as a datum for the tourists' decision-making.

The standard deviation from the mean of each estimated coefficient in Eq. (2) is used to evaluate whether there are statistically significant differences between domestic tourists around the mean coefficient of each destination attribute. If the standard deviation of $\beta_{6n}$ is statistically significant, the sources of heterogeneity between domestic tourists around this visitor attraction category can be identified following Hensher and Greene (2003). Before setting the equation to examine the second hypothesis of this paper (which is focused on the sources of heterogeneity between tourists), tourists' socio-economic and demographic characteristics are analysed as case-specific variables (see Equation 3). The characteristics involved in $S_{ln}$ are gender, marital status, education, age, and tourists' city of origin. The United Cities and Local Governments (UCLG)'s classification of cities (UCLG, n.d.) can be employed to sort the cities of domestic tourists by population size.

(3) $$v_{n}=\underset{l}{\sum }\alpha_{l}\cdot S_{ln}$$

The interaction term between the mean coefficient of $MANMADE_{i}$ and observed domestic tourists' characteristics ($S_{ln}$) in Eq. (4) can serve to identify the characteristics of domestic tourists that explain the differences in domestic tourists' choices of a province around man-made attractions. The estimated coefficients within the category of domestic tourists' city of residence ($\varphi_{9}$ and $\varphi_{10}$) in Eq. (4) are used to examine the second hypothesis of this paper; this is, whether the size of domestic tourists' city of origin is a potential source of taste variation that has an impact on the decision to visit a province with man-made attractions. Case-specific variables are not included separately in Eq. (4) to avoid, first, the significant increase of computational time that occurs when more covariates are added in the mixed logit model (Hensher and Greene, 2003), and second, the inclusion of several estimated coefficients that are not necessary to analyse the research hypotheses of this paper. The no inclusion of case-specific variables in the main equation of a mixed logit model is standard in the literature (Train, 1998).

(4) $$v_{ni}=\delta_{0}+\delta_{1n}DISTANCE_{i}+\delta_{2n}TEMPERA_{i}+\delta_{3n}TEMPERA_{i}^{2}+\delta_{4n}BEACH_{i}+\delta_{5n}MEMORIAL_{i}+\delta_{6n}MANMADE_{i}+\overset{10}{\underset{l=1}{\sum }}\varphi_{l}\left(MANMADE_{i}\cdot S_{ln}\right)$$

If the second hypothesis of this paper is confirmed from the estimation of Eq. (4), then further sources of taste heterogeneity between domestic tourists from different city categories will be captured in part in Eq. (5) and analysed through the estimated coefficients $\gamma_{l,m}$.

(5) $$v_{ni}=\zeta_{0}+\zeta_{1n}DISTANCE_{ni}+\zeta_{2n}TEMPERA_{ni}+\zeta_{3n}TEMPERA_{ni}^{2}+\zeta_{4n}BEACH_{ni}+\zeta_{5n}MEMORIAL_{ni}+\zeta_{6n}MANMADE_{ni}+\overset{10}{\underset{l=9}{\sum }}\phi_{l}\left(MANMADE_{ni}\cdot S_{ln}\right)+\overset{10}{\underset{l=9}{\sum }}\overset{4}{\underset{m=1}{\sum }}\gamma_{l,m}\left(MANMADE_{ni}\cdot S_{ln}\cdot S_{mn}\right)$$

Finally, to determine whether further man-made visitor attractions in a province of destination has an offsetting impact on distance travelled by domestic tourists (the third hypothesis of this paper), an interaction term between $MANMADE_{i}$ and $DISTANCE_{i}$ is added to Eq. (2) as shown in Eq. (6). The direction of the estimated coefficient $\psi_{7}$ is examined for that purpose, which is expected to be positive and statistically significant at the conventional statistical levels.

(6) $$v_{ni}=\psi_{0}+\psi_{1n}DISTANCE_{i}+\psi_{2n}TEMPERA_{i}+\psi_{3n}TEMPERA_{i}^{2}+\psi_{4n}BEACH_{i}+\psi_{5n}MEMORIAL_{i}+\psi_{6n}MANMADE_{i}+\psi_{7}\left(MANMADE_{i}\cdot DISTANCE_{i}\right)$$

To estimate the unknown parameters of Eqs. (2), (4), (5), and (6) with the Mixed Logit model, the maximum simulated likelihood estimator is applied. This is because the integral of logit probabilities over a density of parameters does not have a closed form integration (Train, 2009). Following Hensher and Reyes (2000) and Train (2000), 100 draws are used in this paper to get efficient results. As domestic tourists tend to search for diverse attributes in the destination, correlation patterns between attributes (due to spatiality and cultural complementarity) are allowed in the model. The expected negative coefficient of $DISTANCE_{i}$ is taken as log-normally distributed following studies on recreation demand (see Hynes et al., 2008; Train, 1998). This is consistent with the distance decay theory in that “the demand for any good or service should decline exponentially as distance increases” (McKercher et al., 2008, p. 208). The remaining set of coefficients follow a normal distribution.

### 3.2 Data

Tourists' choices of a province within Colombia for leisure activities were collected from the National Survey of Domestic Tourism Spending, which is conducted by Colombia's Administrative Department of Statistics using a probabilistic, multistage, and stratified sampling method in the main cities of Colombia (DANE, 2014). Data for 3,011 individuals comprise the final sample. Based on this survey, most domestic tourists travel to near destinations, although exception exists; domestic tourists' average length of stay in the destination is 3.21 nights; and their main transport mode to the destination is land transport (92% of domestic tourists travelled by car or bus).

In studies on domestic tourists' choices, provincial destinations or administrative units are usually included as domestic tourists' set of alternatives (Bujosa and Rosselló, 2013; Fuleky et al., 2014; Marrocu and Paci, 2013; Nicolau and Mas, 2006; Priego et al., 2015). This practice is necessary because of the vast number of cities within a country, which becomes difficult to include as a complete set of options of domestic tourists. In this paper, 28 provinces (out of a possible 33 administrative units) are included as domestic tourists' set of alternatives after weighting each destination attribute X in city c by the probability of travelling to that city within province i from tourists' city of origin s ($P_{sc}$). This operation is incorporated in Eq. (7) to capture the relative importance of attribute X within region i for a tourist from city s while eliminating the effect of within-region distance on domestic tourists' choice of city c. A total of 368 cities visited by Colombians are included in Eq. (7). $P_{sc}$ lies between 0 and 1, and $X_{ni}$ is interpreted as the (weighted) average number of attribute *X* in province i.

(7) $$X_{i}=\underset{s}{\sum }\overset{C}{\underset{c=1}{\sum }}X_{c}\cdot P_{sc},\forall c\in i$$

Based on Eq. (7), the attribute $DISTANCE_{i}$ shows the (weighted) average travel distance between tourists' city of origin and province of destination. The attribute $TEMPERA_{i}$ shows the (weighted) average temperature in the province of destination. The attributes $BEACH_{i}$, $MEMORIAL_{i}$, and $MANMADE_{i}$ show the (weighted) average number of available beaches (per square kilometre), the (weighted) average number of memorials alluding to the independence war from Spain (per square kilometre), and the (weighted) average number of man-made attractions (per square kilometre) in the province of destination, respectively. $DISTANCE_{i}$ is calculated employing data from the website http://co.lasdistancias.net; this is a distance calculator provided by distancescalculator.com that has been employed in regional studies (Alfonso, 2017). $TEMPERA_{ni}$ is calculated using statistics from Colombia's Institute of Hydrology, Meteorology and Environmental Studies, and cities' official websites. Statistics on $BEACH_{i}$ and $MEMORIAL_{i}$ are collected from Colombia's official travel website www.colombia.travel.

#### 3.2.1 The latent variable of man-made attractions

The average number of theme parks, gondola lifts, museums, restaurants, and shopping malls (per square kilometre) in each city of Colombia is used to calculate the latent variable $MANMADE_{i}$ through factor analysis. Data on these five attractions are drawn from the Colombian Yellow pages' website https://www.paginasamarillas.com.co (published by Publicar) as there are no consolidated official statistics on the number of man-made attractions in each city of Colombia. The results of the pairwise correlation show a high correlation between these attractions (the correlation exceeds 65%), but not between these attractions and other destination attributes (see Table 1). This finding is fundamental before adopting factor analysis, as the aim of this multivariate analysis is to identify latent variables that capture the correlation between measured variables (Fabrigar et al., 1999).

**Table 1:** Pairwise correlation between provincial attributes.

	DISTANCE	TEMPERA	BEACH	RESTAURANT	MALL	PARK	GONDOLA	MUSEUM	MEMORIAL
***DISTANCE***	1.000								
***TEMPERA***	0.18∗∗∗	1.000							
***BEACH***	0.22∗∗∗	0.31∗∗∗	1.000						
***RESTAURANT***	0.03∗∗∗	0.07∗∗∗	0.22∗∗∗	1.000					
***MALL***	-0.12∗∗∗	-0.06∗∗∗	-0.02∗∗∗	0.76∗∗∗	1.000				
***PARK***	-0.14∗∗∗	-0.09∗∗∗	-0.05∗∗∗	0.66∗∗∗	0.88∗∗∗	1.000			
***GONDOLA***	-0.13∗∗∗	-0.16∗∗∗	-0.12∗∗∗	0.46∗∗∗	0.72∗∗∗	0.82∗∗∗	1.000		
***MUSEUM***	-0.04∗∗∗	-0.05∗∗∗	0.17∗∗∗	0.86∗∗∗	0.79∗∗∗	0.77∗∗∗	0.672∗∗∗	1.000	
***MEMORIAL***	0.04∗∗∗	-0.22∗∗∗	0.14∗∗∗	-0.04∗∗∗	-0.04∗∗∗	-0.08∗∗∗	-0.071∗∗∗	0.013∗∗∗	1.000

Note: these variables are in a continuous scale. ∗∗∗ significant at the 1% critical value.

The presence of a common factor for the group of man-made attractions is confirmed through the principal factor analysis. Table 2 shows that the total variance accounted by Factor 1 (the latent variable $MANMADE_{i}$) is greater than 1 (the Eigenvalue is 3.78), and the difference between Factor1's Eigenvalue and Factor 2's Eigenvalue of 3.38 is high. For Hakstian and Rogers (1982), the substantial drop in the magnitude of the eigenvalue between Factor1 and Factor2 demonstrates the adequacy of including one common factor (Factor1) in this study. Factor1 explains 93.2% of the total variance of the group of man-made attractions for leisure included in this study.

**Table 2:** Factor analysis.

Factor	Eigenvalue	Difference	Proportion	Cumulative
Factor 1	3.786	3.3816	0.9323	0.9323
Factor 2	0.404	0.3650	0.0997	1.0320
Factor 3	0.039	0.1066	0.0098	1.0418
Factor 4	-0.066	0.0362	-0.0164	1.0254
Factor 5	-0.103	.	-0.0254	1.0000

LR test: independent vs. saturated: chi squared (10) = 4.5e+05 *Prob > Chi-squared* = 0.00.

Based on the Kaiser-Meyer-Olkin (KMO) measure of sampling adequacy (Kaiser, 1974) in Table 3, the use of a latent variable $MANMADE_{i}$ is adequate, as the overall proportion of variance among these five man-made attractions explains 78.23% of the variance of $MANMADE_{i}$. The maximum likelihood method is utilised to extract the laten variable, which has desirable asymptotic properties of unbiasedness, efficiency, and normality (Fabrigar et al., 1999).

**Table 3:** Factor loadings and sampling adequacy.

Variable	Factor ($MANMADE_{i}$)	Uniqueness	KMO
Shopping malls	0.9184	0.1565	0.8451
Theme parks	0.9155	0.1619	0.8189
Museums	0.9043	0.1823	0.779
Restaurants	0.8192	0.3289	0.699
Gondola lifts	0.7849	0.3839	0.7604
**Overall**			0.7823

Shopping malls have become an important part of the commercial sector in the cities of Colombia, because shopping malls provide an alternative public space for the joy of people (Grube-Cavers and Carvajal-Sánchez, 2014). People go to shopping malls for shopping (47% of the visitors), for leisure activities (23%), for socialising (9%), for eating (31%), and/or for other reasons (18%) (Grube-Cavers and Carvajal-Sánchez, 2014). Tourists in Colombia have diverse options of theme parks to visit, which have played a significant role in the promotion of tourism since the 1960s (Rivera Rodríguez et al., 2011), as well as museums (MINCULTURA, 2017, November 1) and restaurants with diverse food that show history and culture behind the cities (García and Pardo, 2015). Gondola lifts represent another attraction for domestic tourists in Colombia (Hamón, 2008), especially in the main cities.

#### 3.2.2 Tourists' characteristics as the potential sources of heterogeneity

Domestic tourists' characteristics are included in this study as the covariates that interact with the latent variable $MANMADE_{i}$ to evaluate potential sources of heterogeneity between tourists. Tourists' gender male and female is one characteristic include in this study. Domestic tourists are also classified into the following lifecycle stages based on the age factor: young tourists (between 10 and 30 years of age), adult tourists (between the ages of 31 and 59), and senior tourists (more than 60 years of age). Marital status is separated into married tourists (including living common law), single tourists, divorced tourists (including separated tourists), and widowed tourists. The education level of domestic tourists is divided into university completed, school completed, and school with no graduation (including no school).

The size of tourists' city of residence is taken as a key characteristic that reflects the cities' capacity to provide facilities for leisure activities. Following the United Cities and Local Governments (UCLG)'s classification of cities (UCLG, n.d.), domestic tourists' cities of origin are categorised as intermediary (cities with between 50 thousand and 1 million inhabitants) and metropolitan (cities with more than 1 million inhabitants). Bogotá is classified in this paper as a megalopolis due to its significant size (8 million people, according to statistics from Colombia's bureau of statistics), which is three times the population size of Medellin (the second largest city of Colombia). As noted by Lang and Knox, “…a key element of the new metropolis is its vast scale, which facilitates the emergence of a trans-metropolitan urban structure –the megapolitan region” (Lang and Knox, 2009, p. 789). There is no classification of small cities in this study, as the sample of tourists' city of residence does not include small cities.

A descriptive analysis in Table 4 shows important facts that will be used later to evaluate the second hypothesis of this paper; this is, whether there are taste differences between domestic tourists around the mean coefficient of man-made attractions that can be explained by the size of domestic tourists' city of origin. On average, 53.5% of domestic tourists prefer cities whose number of man-made attractions is above the average of the province visited. However, the average probability for domestic tourists from intermediary and metropolitan cities (52.5% and 56%, respectively) are higher than the probability for domestic tourists from Bogotá (51.9%). These figures suggest that, compared to tourists from Bogotá, the tourists from metropolitan and intermediary cities are more motivated to visit provinces with a relatively higher number of man-made attractions (above the provincial average). Of tourists from metropolitan and intermediary cities, the representative tourists from metropolitan cities are the most motivated to visit provinces with more man-made attractions for leisure and recreation.

**Table 4:** Domestic tourists' preferences for cities with man-made attractions.

City category	Preferences (%) for cities with man-made attractions compared to the provincial average	
	Above provincial average	Below provincial average
Intermediary	52.5	47.5
Metropolitan	56.0	44.0
Bogotá	51.9	48.1
Total average	53.5	46.5

Note: the addition of values in each row equals 100%.

#### 3.2.3 Man-made attractions and its moderating role of distance

Previous statistical analyses of the data used in this paper show that the probability of travelling to a regional destination decreases exponentially as the travel distance from tourists' city of origin increases. Table 5 includes a descriptive analysis on the extent to which man-made attractions can lessen the negative effect of distance on tourists' choices. This analysis will be linked later in the empirical results section to the third hypothesis of this paper. The figures show domestic tourists' probability of travelling to a city where the number of man-made attractions is above or below the average number of man-made attractions in the province visited. The visited city can be in tourists' own province, in a contiguous province, or in a non-contiguous province.

**Table 5:** Tourists' preferences for cities with man-made attractions by provincial categories.

City category	Preferences (%) for cities with man-made attractions compared to the provincial average, by provincial category					
	Own province		Contiguous province		Non-contiguous province	
	Above aver.	Below aver.	Above aver.	Below Aver.	Above Aver.	Below Aver.
Intermediary	14.0	27.8	17.8	11.2	20.7	8.5
Metropolitan	16.3	27.8	26.3	7.1	13.4	9.2
Bogotá	11.8	27.9	21.8	11.1	18.3	9.2
Total average	14.0	27.8	22.0	9.8	17.4	8.9

Note: the addition of values in each row is equal to 100%.

The figures also show that the average domestic tourist is not generally driven by man-made attractions when he/she travels within his/her own province (the probability is 14%). When the tourists travel to a province that is contiguous to his/her own province, their preferences are for cities where the number of man-made attractions is above the regional average (the probability is 22%). This is more evident for tourists from metropolitan cities, as 26.3% of these tourists reported to travel to cities located in contiguous provinces, where the number of man-made attractions is above the provincial average. This pattern of preferences remains when the tourist travels to a city located in a province that is non-contiguous to his/her own province, although the tourists' preferences for such cities are lower than their preferences for cities located in contiguous provinces (the probability is 17.4%).

#### 3.2.4 Summary statistics

The summary of statistics on destination attributes is presented in Table 6. The average distance between tourists' city of origin and his/her destination is 167 km. The mean temperature of the cities visited is 24 degrees Celsius. The average number of man-made attractions in the cities visited is 24.6, or 0.07 attractions per square kilometre. There are destination cities without man-made attractions, while Bogotá (the main city of Colombia) has more than two thousand man-made attractions. There is an average number of 0.12 beaches available in the cities visited, and 0.008 war memorials alluding to the independence war from Spain.

**Table 6:** Summary statistics on destination attributes.

Attributes	Mean	Std. Dev.	Minimum	Maximum
*DISTANCE (km)*	167	167	6.4	1,204
*TEMPERA*	24.18	4.75	11.6	33
*MANMADE*	24.6	163.11	0	2,289
*MANMADE/km*^*2*^	0.07	0.46	0	6.02
*BEACH*	0.125	0.83	0	12
*MEMORIAL*	0.008	0.116	0	2

Note: the mean value of each attribute shows the average value calculated for 368 cities visited by domestic tourists.

Statistics on the characteristics of domestic tourists in Colombia (Table 7) show that most tourists who travel for leisure activities are female (55%). The majority are young tourists (48%) and adults (42%). Most tourists are single (46%), followed by divorced (28%), married (22%), and widowed (4%). Regarding education, 53% of tourists have completed university studies, 22% have completed secondary school, and the remaining 25% have not completed secondary school, or do not have years of schooling. Most tourists are from intermediary cities (55%) and metropolitan cities (30%).

**Table 7:** Statistics on the characteristics of domestic tourists.

Characteristics	Obs.	Percentage	Characteristics	Obs.	Percentage
Male	1355	45	Widowed	117	4
Female	1656	55	University completed	1603	53
Young	1452	48	School completed	647	22
Adults	1259	42	School no completed/no School	761	25
Seniors	300	10	Intermediary city	1664	55
Single	1394	46	Metropolitan city	888	30
Divorced	841	28	Bogotá	459	15
Married	659	22			

Note: Percentage shows the percentage share of domestic tourists containing each characteristic.

A summarised analysis of domestic tourists' choices amongst regional destinations from Eq. (3) is also carried out based on tourists' characteristics. In total, 270 coefficients are estimated from 27 alternatives (excluding the control group) and 10 socioeconomic and demographic characteristics. Key results show that *metropolitan* and *intermediary* cities have the highest frequency of statistically significant values, suggesting that the size of tourists' city of origin (a proxy variable of the capacity of residents' city of origin to supply venues for locals and tourists) is a fundamental factor to include in the study. Overall results show statistically significant regional choice differences between tourists from *metropolitan* and *intermediary* cities (compared to tourists from Bogotá) in 70% of the regional alternatives chosen by these tourists.

## 4 Empirical results and discussion

The estimates of the Mixed Logit model in Eq. (1) that includes the domestic tourists' observed utility of Eqs. (2) and (4) to (6) are reported in Table 8. Analyses are presented in two analytical parts. The first part shows the results of Eq. (2) (column (i)) to analyse the mean effect of each regional attribute on domestic tourists' choices, and the standard deviation from the mean. The coefficient of $MANMADE_{i}$ is used to examine whether domestic tourists' likelihood of choosing a province increases if the average number of man-made attractions rises. The standard deviation of $MANMADE_{i}$ is employed to evaluate whether there is heterogeneity between domestic tourists around the mean coefficient of man-made attractions. The second part shows the results of Eqs. (4) and (5) (columns (ii) and (iii)) to identify possible sources of heterogeneity between domestic tourists around the mean coefficient of $MANMADE_{i}$, including the tourists' city of origin. This second part also shows the results of Eq. (6) (column (iv)) to analyse the hypothesised moderating role of man-made attractions on the effect of distance on domestic tourists' choices.

**Table 8:** Estimation results of domestic tourists' destination choices in Colombia.

Dependent variable: domestic tourists' likelihood of travelling to a province ($P_{ni}$)								
Independent variables:			Interaction terms of $MANMADE_{i}$					
	Baseline Eq. (2)		Covariates Eq. (4)		Covariates Eq. (5)		Covariate Eq. (6)	
	Coef.	Std. error	Coef.	Std. error	Coef.	Std. error	Coef.	Std. error
	(i)		(ii)		(iii)		(iv)	
**Mean**								
$DISTANCE_{i}$	-4.403∗∗∗	0.045	-4.445∗∗∗	0.046	-4.430∗∗∗	0.046	-0.013∗∗∗	0.000
$TEMPERA_{i}$	0.615∗∗∗	0.125	0.508∗∗∗	0.144	0.436∗∗∗	0.113	0.443∗∗∗	0.110
$TEMPERA_{i}^{2}$	-0.012∗∗∗	0.002	-0.010∗∗∗	0.003	-0.009∗∗∗	0.002	-0.009∗∗∗	0.002
$BEACH_{i}$	0.224∗∗∗	0.022	0.225∗∗∗	0.022	0.223∗∗∗	0.022	0.171∗∗∗	0.019
$MEMORIAL_{i}$	0.171	0.359	0.071	0.349	-0.139	0.396	0.175	0.320
$MANMADE_{i}$	0.193∗∗∗	0.038	-0.086	0.120	0.023	0.074	-0.206	0.182
**Standard Deviation**								
$DISTANCE_{i}$	1.456∗∗∗	0.061	1.748∗∗∗	0.072	1.441∗∗∗	0.061	0.009∗∗∗	0.000
$TEMPERA_{i}$	0.806∗∗∗	0.100	0.894∗∗∗	0.133	0.900∗∗∗	0.121	0.873∗∗∗	0.110
$TEMPERA_{i}^{2}$	0.014∗∗∗	0.002	0.015∗∗∗	0.002	0.015∗∗∗	0.002	0.016∗∗∗	0.002
$BEACH_{i}$	0.101∗∗∗	0.034	0.103∗∗∗	0.031	0.08∗∗∗	0.042	0.184∗∗∗	0.027
$MEMORIAL_{i}$	2.496∗∗∗	0.513	2.334∗∗∗	0.462	2.780∗∗∗	0.524	1.787∗∗∗	0.423
$MANMADE_{i}$	0.227∗∗∗	0.042	0.151∗∗∗	0.038	0.216∗∗∗	0.045	0.137∗∗∗	0.049
**Interaction variables with**$\mathrm{MANMAD}E_{i}$								
Male			-0.0024	0.037				
Young			0.0535	0.090				
Adults			0.0534	0.082				
Married			-0.0165	0.112				
Single			0.0357	0.115				
Divorced			0.0139	0.108				
School−completed			0.0755	0.055				
University−completed			0.0408	0.047				
Metropolitan			0.264∗∗∗	0.072				
Intermediary			0.183∗∗∗	0.066				
Metropolitan⋅Male					0.044	0.067		
Metropolitan⋅Adults					0.063	0.072		
Metropolitan⋅Married					-0.195∗∗	0.092		
Metropolitan⋅								
School−completed					0.024	0.068		
Intermediary⋅Male					-0.011	0.047		
Intermediary⋅Adults					0.005	0.049		
Intermediary⋅Married					0.055	0.058		
Intermediary⋅								
School−completed					-0.003	0.048		
Metropolitan					0.226∗∗	0.093		
Intermediary					0.168∗∗	0.075		
$DISTANCE_{i}$							0.095∗∗∗	0.0364
$MSL\left(\tilde{\theta }\right)$	-6002		-6005		-6003		-6040	

∗∗∗significant at 1%, ∗∗significant at 5% and ∗significant at 10%.

Note: the coefficient of $DISTANCE_{ni}$ is log-normally distributed in Eqs. (2), (4), and (5); it is normally distributed in Eq. (6).

Following McFadden and Train (2000), the likelihood ratio test (LR test) is applied to know the adequacy of using the Mixed Logit model as compared to the Conditional Logit model (not reported). At the 1% level of significance, the null hypothesis that the Conditional Logit model is nested in the Mixed Logit model is rejected. Therefore, the inclusion of random parameters in the Logit model substantially improves the model fit as compared to the standard Conditional Logit model. A robustness check is also applied to the estimated results from Eq. (2) (column (i)) following Lu and White (2014) and Barslund et al. (2007). The core variables selected for this check are *DISTANCE*, *TEMPERA, TEMPERA*^*2*^, and *MANMADE*. Although the selection of these variables can be subjective, we focus on the attributes that are present in most of the regional alternatives. The secondary (testing) variables are *BEACH* and *MEMORIAL*. In total, four regressions are estimated: i) only the core variables; ii) the core variables and *BEACH*; iii) the core variables and *MEMORIAL*; and iv) all variables (core and secondary). The results suggest that the core variables are robust. The estimated coefficients of all variables have the same expected sign in all regressions and are statistically significant (only the temperature variable turns out to be sensitive to the inclusion of the variable *BEACH*).

### 4.1 The effects of regional attributes on domestic tourists' choices

Based on the mean coefficient of $DISTANCE_{i}$ in column (i), there is statistical evidence to suggest that the average domestic tourist in Colombia prefers closer destinations. This result confirms the negative effect of distance on domestic tourists' choices in Colombia; a result also found by Nicolau and Mas (2006), Monfort et al. (2010), and Álvarez-Díaz, D'Hombres, and Ghisetti (2017) in Spain; by Huybers (2003) in Australia; and Marrocu and Paci (2013) in Italy. This outcome cannot be generalised for all domestic tourists, however, as their individual standard deviation from the mean is statistically significant. Some domestic tourists tend to prefer closer destinations to those that are further away, and vice versa. As noted by Baxter (1979) in recreational demand, longer distances are sometimes preferred by people as the trip to a distance destination can give satisfaction by itself.

The mean coefficient of $TEMPERA_{i}$ in column (i) is positive and statistically significant and suggests that the representative Colombian tourist prefers warmer temperatures to cooler ones. A statistically significant turning point is found at 23.7 degrees Celsius, which indicates that for provinces with an average temperature above this, tourists are less willing to travel there. This result is consistent with Bujosa and Rosselló (2013) and Muñoz et al. (2021) for domestic tourism in Spain, who found that temperature is an important factor at the time of travelling to a destination, although there are differences in the effects of weather on tourism amongst the year's seasons. Muñoz et al. (2021) also found that a strong temperature fluctuation in the destination leads to a decline in tourism demand. Eugenio-Martin and Campos-Soria (2010a) found that the European households' probability of travelling domestically, compared to the willingness of travelling abroad, increases as the climate in the region of residence improves (measured through a tourism climate index that includes the average temperature of the region). The statistically significant standard deviation of $TEMPERA_{i}$ in column (i) shows taste differences between domestic tourists in Colombia, however, indicating that warm temperature in the tourist destination is important for a group of residents, while for another group of tourists, it is not the case.

Table 9 extends the temperature analysis using crosstab tables and shows that 83.4% of the tourists who travel from cities with an average temperature between 12 and 16 degrees Celsius prefer cities of warmer temperatures. The percentage preferring destinations with warmer climate reduces to 34.6% for tourists who travel from cities with a 19-to-23 degrees Celsius temperature range. And 78.9% of tourists who travel from cities with a temperature between 25 and 28 degrees Celsius prefer to visit destinations with similar temperatures.

**Table 9:** Domestic tourists' preferences for colder, similar, and warmer Cities.

Average Temperature in the city of origin (degrees Celsius)	Temperature in the city of destination		
	Warmer	Similar	Colder
12–16	83.43%	15.92%	0.65%
18–23	34.65%	51.82%	13.53%
25–28	0.69%	78.90%	20.38%

Note: to define whether a city of destination is Colder, Similar or Warmer than domestic tourist's city of origin, 4 degrees Celsius were added/subtracted from the temperature recorded in domestic tourists' city of origin. This value of 4 was obtained from the standard deviation of the temperature recorded in domestic tourists' city of destination.

Domestic tourists' likelihood of travelling to a province increases if the comparative number of beaches per square kilometre ($BEACH_{i}$) increases (column (i) in Table 8). Beaches is an important nature-based attraction for the average domestic tourist in Colombia, although there is heterogeneity between them. Álvarez-Díaz et al. (2017) and Marrocu and Paci (2013) found a positive relationship between domestic tourists' origin-destination flows and beaches in Spain and Italy, respectively. De-la-Mata and Llano-Verduras (2012) found changes in domestic tourists' preferences for non-coastal destinations in Spain after a 7-year period.

The mean coefficient of $MEMORIAL_{i}$ is positive, although is not statistically significant (column (i) in Table 8). The standard deviation of the coefficient of the same attribute is significant and large, however. This conjoint result implies that the average number of memorials commemorating the independence war from Spain in Colombia's regions influence the choices of domestic tourists. Some residents opt for provinces with war memorials while other residents do not. The mean is not significantly different from zero because domestic tourists' differences regarding war memorials tend to balance out in the population. As found by Priego et al. (2015) and Patuelli et al. (2013) for the Spanish and Italian case, respectively, domestic tourists' trip to a province is influenced by the presence of World Heritage Sites. However, the presence of these sites in tourists' region of origin tends to discourage their trip to other destinations (Patuelli et al., 2013). Giambona and Grassini (2020) found that historical and cultural resources are fundamental to attract domestic tourists in Italy.

Key results show that the mean coefficient of $MANMADE_{i}$ is statistically significant at the 1% significance level (column (i) in Table 8), suggesting that the average domestic tourist in Colombia prefers provinces with more man-made attractions. This result is consistent with the study by Marrocu and Paci (2013) and Hearne and Salinas (2002), who found that quality museums and restaurants, and aerial trams, respectively, are venues that influence domestic tourists' trip to a destination. Álvarez-Díaz et al. (2017) found that, in the Spanish case, there is enough evidence on the positive contribution of theme parks to the inter-regional tourism flows, although not for the museums' case. Based on the standard deviation of the coefficient of $MANMADE_{i}$ in Table 8 (column (i)), it is possible to evidence differences between domestic tourists around the average number of man-made attractions in the destinations at the 1% significance level. This suggests that while a group of tourists prefers to visit regions with a relatively high number of man-made attractions (above the regional average), another group prefers a relatively small number of these attractions (below the regional average).

An analysis of marginal effects is carried out to estimate how much the probabilities of selecting a regional alternative change if the factor score of man man-made visitor attractions increases by one unit (per squared kilometre). The delta method for the calculation of standard errors is used. The results show an average increase in the probability of choosing a regional destination due to a unit increase in the score of man-made venues of 0.25 percentage points (pp), excluding nonsignificant results. Twenty-three out of 28 provincial destinations included show positive and statistically significant marginal effects from an increase in man-made attractions. Provinces with the greatest percentage point increase being statistically significant are in the Andean region (Antioquia (0.64 pp), Valle del Cauca (0.47 pp), Caldas (0.46 pp)) and the Caribbean region (Bolivar and Magdalena (0.44 pp each)). Provinces with the lowest percentage point increase are in the Amazon region (Putumayo (0.017 pp) and Amazonas (0.0034 pp)) and the Orinoquía region (Arauca (0.0068 pp)). These three last provinces, which hold the lowest relative frequencies of visits by domestic tourists within the analysed sample, have a low population density and a comparatively low number of man-made venues for visitors' leisure.

### 4.2 Tourists' city of origin as a source of heterogeneity

This second part analyses the potential sources of heterogeneity between domestic tourists around the average number of man-made attractions (columns (ii) and (iii) of Table 8). The statistically significant coefficients for *Metropolitan* and *Intermediary* of 0.264 and 0.183, respectively, in Column (ii) confirms that tourists' city of origin is one of this sources of heterogeneity. Tourists from metropolitan and intermediary cities are more motivated than tourists from Bogotá to visit provinces where more man-made attractions exist. This is expected, as very large cities like Bogotá (with more than 8 million inhabitants) have plenty of man-made attractions compared to smaller cities. The number of man-made attractions in Bogota is 1.74 and 11.2 times the average number recorded in metropolitan and intermediary cities, respectively. These results complement the descriptive analysis presented in section 3.2.2 using statistical methods and shows the significance of addressing adequate marketing strategies toward the attraction of tourists motivated by man-made venues in the destination based on the size of tourists' city of origin.

Further sources of taste heterogeneity between domestic tourists from metropolitan and intermediary cities are explored in column (iii) (Table 8). The estimated result for *Metropolitan∙Married* coefficient is negative (-0.195) and significant at the 5% level. This finding is in line with Kaynak et al.’s (1996) study, who found that single people search for exciting experiences in the destination, as well as fun and nature activities, while married visitors are more oriented toward restful and refreshing activities. Based on Nicolau (2010), the desire of seeking variety through man-made attractions tends to be higher for single and divorced tourists than for married visitors. Therefore, the construction of man-made venues for leisure as a strategy to attract tourists from metropolitan cities should be more oriented toward single, divorced, and widowed tourists than married tourists. Other estimated results in column (iii), Table 8, show insufficient statistical evidence of further sources of taste heterogeneity.

### 4.3 The moderating role of man-made attractions on distance effects

The sign of the coefficient for $MANMADE_{i}\cdot DISTANCE_{i}$ of 0.095 in column (iv), Table 8, shows evidence to infer that increases in the average number of man-made visitor attractions in the destination lessen the mean effect of distance on domestic tourists' preferences in Colombia. This finding complements the descriptive analyses presented in sub-section 3.2.3 and suggests that the construction of venues for leisure can be an important strategy to attract more domestic tourists to distant destinations (usually poor regions in Colombia), and therefore, increase tourism consumption and employment in those regions. The result also complements the studies that have identified motivations that moderate the effect of distance on domestic tourists' choices, including climate and tranquillity (Nicolau and Mas, 2006).

These results are of fundamental interest for tourism stakeholders, including regional authorities, as the findings show that the strategies to attract tourists through the construction/enhancement of man-made venues for leisure and recreation should be planned based on the tourists' city of residence; this is, based on the capacity of residents' city of origin to supply venues for leisure and recreational activities, and based on the travel distance to get to the regional destination from the tourists' city of origin.

## 5 Concluding remarks

This paper analyses the influence of provincial attributes in Colombia to attract domestic tourists using the Mixed Logit model. The study focuses on the role of man-made attractions for leisure and recreation on domestic tourists' preferences amongst regional alternatives. A latent variable of man-made attractions is inferred from a group of venues that attract visitors, such as museums, restaurants, aerial trams, theme parks, and shopping malls. The findings first show that the likelihood of travelling to a destination by domestic tourists increases as the number of man-made attractions for leisure and recreation (per square kilometre) rises. This result can be generalized to other countries (where the supply of man-made venues for leisure can potentially play a significant role in the demand for tourism) and indicates the importance of regional strategies to build and/or enhance man-made venues to attract locals and tourists.

The paper, however, found significant sources of taste heterogeneity between domestic tourists around the cluster of man-made attractions for leisure and recreation. Results on domestic tourists' preferences suggest that the construction and/or enhancement of venues for leisure activities should be primarily done in tourism destinations that attract people from smaller cities (metropolitan and intermediary cities, respectively) compared to Bogotá (the megalopolis of Colombia). This finding can be extended to several country cases, as tourists' choices of a destination that provides man-made venues for leisure are likely to differ between tourists. Strategies to attract domestic tourists from metropolitan cities through man-made attractions should be more focused on single, divorced, and widowed domestic tourists, as this group of tourists tends to be more oriented toward made-made attractions compared to married domestic tourists.

Finally, the capacity of man-made attractions to moderate the negative effects of distance on domestic tourists' choices is also analysed in this study. The evidence shows that an increase in the number of man-made attractions in a destination tends to moderate the negative effect of distance on tourists' preferences for that destination. Thus, the construction and/or enhancement of tourism facilities can serve as a strategy to increase domestic tourism trips to distant destinations. This result can also be evident in other countries, although the generalisation could depend on other regional factors associated with tourists' decision-making, including climate, prices, risk levels in the destination, amongst other attributes. These distant destinations are in many cases economically lagging areas that pose plenty of attributes to attract tourists. The strategies in Colombia can target more intensively domestic tourists from metropolitan cities, when the city of destination is in a contiguous province, and from intermediary cities when the destination city is in a non-contiguous province.

A limitation identified in this study is the inability to include tourism prices in the set of tourism destinations (due to data unavailability) and domestic tourists' income (the survey does not include such variable) as factors that also determine domestic tourists' choices. Recent domestic tourism surveys are including statistics on tourists' income, which could be used in future analyses. The use of cross-section surveys (rather than longitudinal data) is also a limitation to this study. Future studies could include information on the quality of man-made attractions from reviews on Tripadvisor, Trivago, and other platforms, and could also identify other factors that can potentially offset the negative effect of distance on tourists' choices. Finally, a study on the influence of destination attributes on international tourists' preferences could also be realised following this paper to identify adequate strategies for each market segment separately.

## Declarations

### Author contribution statement

Andres Camacho-Murillo: Conceived and designed the experiments; Performed the experiments; Analyzed and interpreted the data; Contributed reagents, materials, analysis tools or data; Wrote the paper.

Rukmani Gounder and Sam Richardson: Conceived and designed the experiments; Analyzed and interpreted the data; Wrote the paper.

### Funding statement

This work was supported by Colombia’s Ministry of Science, Technology, and Innovation (Minciencias), agreement 646/2014.

### Data availability statement

Data associated with this study has been deposited online at OSF (https://osf.io/x4t58/).

### Declaration of interests statement

The authors declare no conflict of interest.

### Additional information

No additional information is available for this paper.
